# Supplementary material for: Are carbon emissions trading and green financial instruments synergistic? -Comprehensive quantitative research based on content analysis
Source: PLoS One. 2024 Mar 7;19(3):e0298601. doi: 10.1371/journal.pone.0298601 (PMC10919601; doi:10.1371/journal.pone.0298601)
Supplement: S1 Data — (ZIP) [file pone.0298601.s001.zip › Data/地级市Z_F工作报告.zip!/║╙─╧/╨┬╧τ╩╨/└┤╫╘╣1⁄2╓┌║┼í╛┬φ┐╦╩2╛▌═°í┐_2016.docx]

各位代表：

　　现在，我代表市人民政府，向大会作工作报告，请予审议，并请各位政协委员和其他列席人士提出意见。

　　一、2015年及“十二五”工作回顾

　　2015年是“十二五”的收官之年，市人民政府在省委、省政府和市委的坚强领导下，在市人大、市政协的监督支持下，团结带领全市人民，把稳增长作为全局工作的突出任务，围绕关键环节发力，有力促进了经济逐步回暖。全年完成地区生产总值1982.3亿元，总量位居全省第6位，同比增长6%，增速较一季度、上半年分别提高1.9和0.9个百分点；规模以上工业增加值同比增长5.8%，增速较一季度、上半年分别提高2.3和1.1个百分点；一般公共预算收入144.6亿元，总量位居全省第4位，同比增长4%，增速较一季度、上半年分别提高6.8和1个百分点。主要做了以下工作：

　　（一）狠抓关键环节，全力稳定增长。针对影响经济运行的重点领域，综合施策，全力稳增长、保态势。一是稳定工业运行。由市级领导分包重点企业，百户重点企业反映的152个问题已基本解决到位。1133家规模以上工业企业中，336家大中型企业增加值同比增长8.4％，拉动全市工业增加值增长6.5个百分点。二是稳定房地产市场运行。出台扶持房地产发展的二十二条意见和支持农民进城购房等政策，房地产销售面积、投资增速分别较一季度提高29.7和21.5个百分点，市区二手房交易面积同比增长94.5%。三是扩大有效投资。签约亿元以上项目242个，同比增加93个；总投资1109亿元，同比增长17.2%。总投资30亿元的华兰生物单抗生产基地、20亿元的白鹭化纤10万吨绿色纤维等199个亿元以上项目开工建设。招商项目合同履约率、资金到位率、开工率同比分别提高4.9、18.4和11.3个百分点。四是强化要素保障。4次银企洽谈会共签约139亿元，到位94亿元。获批建设用地3.06万亩，同比增长30%，新获批“人地挂钩”批次建设用地3677亩，通过人地挂钩上报的5435亩用地已全部获批。

　　（二）坚持问题导向，逐步破解难题。从制约发展的突出问题入手，加强顶层设计，逐步破解发展瓶颈。在金融领域，出台金融化险“1+6”政策体系，建立产业基金、PPP模式跟进、主办银行制度、加快企业上市等系列顶层设计，整体谋划推动金融业发展。企业应急转贷资金规模增加至3亿元，全年完成转贷27笔，累计转贷4.7亿元。组建全省首个市级新型城镇化发展基金，首期注册100亿元，创新“基金+”模式，有效破解了基础设施建设资本金不足的难题。与深创投设立首期规模1亿元的创业投资基金，已与4家企业达成合作协议。与沪江网合作的首期3000万元“互联网+”蚂蚁雄兵基金已完成核准注册，即将开始运行。与省科技厅合作的科技创新风险投资基金正在积极洽谈。推行主办银行制度，建立公共资源存贷激励机制，鼓励银行贷款向实体经济倾斜，处置企业互保风险，79家市重点企业及64家县（市）、区企业与金融机构签订主办银行合作协议，涉及贷款227亿元。新增新三板挂牌企业9家，累计达14家，融资3.2亿元，挂牌企业数量位居全省第3位。处非工作总体趋稳向好，追回赃款赃物价值近20亿元。在环境保护领域，狠抓大气污染防治，适时限产一批企业，坚决关闭一批污染严重的企业；拆除155台10蒸吨以下燃煤小锅炉；淘汰黄标车7995辆，排名位居全省第4位。推进水污染治理，完成5家污水处理厂改扩建工程，铁西北污水处理厂开工建设，全市日污水处理能力达84.5万吨，共产主义渠自8月份开始全面达标，卫河自11月底实现达标。在城市建设和管理领域，把棚改例会延伸到项目现场召开，重点推进的公村等15个城中村（棚户区）改造项目，累计拆迁200万平方米；启动生态水系建设，赵定河全线综合治理工程正式启动，完成投资1400万元；定国湖工程在4个月内完成前期筹备工作并正式启动，目前土方开挖已完成30%。交通工作负重前行，省政府解除对我市交通项目限批，新晋高速、鹤辉高速推进步伐加快，平原路东延完成总工程量的77%。建立城市管理“1+4”机制，加强问题督察治理，有力保障了全国文明城市创建等工作开展，全国文明城市首个年度的创建工作取得全国同类城市第9名的好成绩。

　　（三）推动产业转型，强化发展支撑。推进外引内联，加快兼并重组，培育产业集群，提升竞争力。一是完善规划，培育集群。确立产业发展“4325”工程，把产业集群作为主攻方向，新签约的242个亿元以上项目中，产业集群关联项目达128个。在全省产业集聚区观摩活动中，原阳县产业集聚区代表我市夺回豫北片区第1名。借助落后产能淘汰，加快企业重组整合，8户企业完成兼并重组，10户正在推进，有重组意向的44户。中航工业新飞集团以6500万欧元收购欧洲冷藏车行业龙头法国兰博瑞特公司；新航集团收购德国海力达后，2015年主营业务收入突破百亿、达到102亿元。二是夯实基础能力，发展现代农业。建成高标准粮田50.8万亩，粮食总产达43亿公斤，同比增长3.4%。销售收入10亿元以上的农业产业化集群达15个。“新乡小麦”成为国家地理标志农产品。创建高效农业园区50个。新增土地流转面积13万亩。三是借助“两区”等载体，大力发展服务业。商务中心区和特色商业区完成投资35亿元。旅游业接待国内游客人数、实现国内旅游收入同比分别增长12.5%和15%。宝泉和轿顶山景区成功创建国家4A级景区。

　　（四）加快创新创业，转换发展动力。把创新作为引领发展的第一动力，培育引进新业态，推动大众创业、万众创新。一是推进科技创新。出台科技创新“1+5”政策体系，举办“科技支撑区域经济发展——走进新乡技术专题对接会”，浙江大学等48家省内外知名高校参会，11家单位与知名高校、科研院所签署对外科技合作项目。组建科技协同创新创业联盟，新培育省级以上企业研发中心15家，新增国家高新技术企业17家、总数达81家，专利申请量突破4000件，4个项目获得国家科技进步二等奖。二是发展新业态。引进“互联网+”等新业态项目76个，总投资72亿元；电子商务交易额103亿元，同比增长21%；高新区成功创建省级电子商务示范基地；与沪江网在“互联网+教育”等领域开展深度合作。三是推进大众创业。封丘县电商产业园入驻企业42家，集聚创业团队35个；红旗区国家863科技产业园众创空间入驻企业36家、创业团队25个；高新区电子信息科技园入驻企业25家、创业团队30个；新乡县与阿里巴巴农村淘宝网签署合作协议，第一批37个村级服务站运行，营业额达1840万元；经开区互联网外包孵化基地进驻电商企业10家；科隆集团组建全市第一家创客中心。新登记市场主体3.7万户，同比增长18.4%。四是深化各项改革。落实上级改革部署，抓好简政放权、公车等43大类改革任务落实，各项年度改革任务基本完成。

　　（五）持续改善民生，维护社会稳定。全年各项民生支出232.4亿元，同比增长10.9%，占公共财政支出的74.9%。城镇居民和农民人均可支配收入分别达25349元和11772元，同比分别增长8%和8.5%。一是推进精准扶贫。实现5.36万农村贫困人口脱贫。黄河滩区居民迁建试点工程全面启动，第一批647户群众已搬迁入住。二是提升就业能力。城镇新增就业9.62万人，失业人员再就业2.86万人，农村劳动力转移就业125.9万人次，城镇登记失业率3.98%。三是加强社会保障。城镇基本医疗保险参保人数增加1.6万人。城乡居民基本养老金由每人每月60元提高至78元以上。竣工分配公共租赁住房33466套。拨付财政补助资金4亿元，对城乡低保户、农村五保户实施救助。为劳动者追讨工资2698万元，其中农民工工资2462万元，涉及农民工3306人。四是发展教育事业。积极推进义务教育均衡发展，本科上线率达51.88%，高出全省9.28个百分点；接收进城务工人员随迁子女入学10883人，实现“应入尽入、同城同待遇”。河南师范大学成为全省第3所与教育部共建的高校；河南机电高等专科学校申报设置河南工学院工作目前已通过教育部公示。五是提升群众健康水平。134个中央投资卫生项目顺利实施，市中心医院东区医院主体建筑封顶。新农合人均筹资标准由380元提高到470元，财政补助标准人均提高60元，参合率达99.69%。六是推进各项事业协调发展。南水北调配套工程6月底实现通水，目前南水北调水已占市区居民用水量的75%。解决了28.7万农村人口和学校师生的饮水安全问题。“单独两孩”生育政策平稳实施。全国质量强市示范城市创建工作稳步推进。加强国防动员和后备力量建设，全国“双拥”模范城创建顺利通过验收。安全生产、信访稳定等工作有效推进，民族团结、宗教和睦局面不断巩固，应急管理能力逐步增强，社会大局和谐稳定。

　　一年来，我们坚持依法行政，自觉接受人大依法监督、政协民主监督和社会各界监督，办理省市人大代表建议201件、政协委员提案505件，办结率100%。深入开展“三严三实”专题教育，治理懒政怠政为官不为，共查处案件83起，给予党政纪处分95人。强化权力运行监督制约，“三公”经费同比下降24%。人民防空、统计审计、新闻出版、外事侨务、史志档案、气象地震等工作都取得新成绩。

　　一年来，我们还积极争取上级支持，新获得一批国家级支持平台。被国家发改委确定为产业集聚集群创新发展综合改革试点，成为全国5个综合改革试点之一；被国家发改委、财政部、住建部联合确定为国家循环经济示范城市建设地区；郑洛新国家自主创新示范区已报国务院待批；被工信部评为宽带中国示范城市、国家信息消费试点城市；被科技部批准为第六批国家农业科技园区；被国家质检总局评为10个“全国质量魅力城市”之一。这些荣誉的获得，不仅有效提升了我市整体形象，也为下步工作开展搭建了广阔平台。

　　各位代表!

　　2015年工作取得了积极成就，标志着“十二五”规划确定的主要任务圆满完成。过去5年，全市上下迎难而上、顽强拼搏，各项事业取得重大成就。一是综合实力显著提升。地区生产总值由“十一五”末的全省第九位上升到第六位；公共财政预算收入总量翻番；全社会固定资产投资完成近8000亿元，是“十一五”时期的2倍。二是产业结构持续优化。二三产业比重达88.8%，比“十一五”末提高1.8个百分点。粮食总产、单产均实现“十二连增”；规模以上工业产值超过4000亿元，年均增长13.1%；服务业增加值比重较“十一五”末提高7.1个百分点。三是发展后劲不断增强。实施亿元以上项目1526个，完成投资4750亿元；新乡高新技术产业开发区、新乡经济技术开发区晋升为国家级园区，产业集聚区工业增加值、固定资产投资分别占全市的63.6%和51%。四是城乡面貌明显改善。常住人口城镇化率达49%，高于全省2.15个百分点。市区棚户区（城中村）改造在建项目达51个，铁西区域开发和大东区规划建设正式启动。以市区为中心、平原示范区为副中心、县城、重点乡镇为支撑的城镇发展体系初步建立。五是发展活力有效释放。简政放权力度加大，行政审批事项由469项减少至135项；实际利用外资37亿美元，是“十一五”的3.3倍。企业技术研发中心达624家，其中省级以上243家，位居全省第二位。各类市场主体达16万家，比“十一五”末增长39.4%。

　　各位代表!

　　“十二五”以来，特别是2015年，我们面临的大事多、难事多，全市上下克难攻坚、奋力进取，逐步解决了一批深层次矛盾，取得了来之不易的成果，积累了在困难中前进、在竞争中发展的宝贵经验。这是省委、省政府和市委坚强领导的结果，是全市人民共同奋斗的结果。在此，我代表市人民政府，向在各个岗位上付出辛勤汗水的全市人民，向给予政府工作大力支持的人大代表、政协委员，向各民主党派、工商联、人民团体和各界人士，表示崇高的敬意！向驻新官兵、驻新单位及所有关心支持新乡发展的朋友们，表示衷心的感谢！

　　我们清醒认识到，政府工作还存在诸多不足：面对新常态、新形势，一些单位思想不够解放，敢闯敢试、敢于担当的劲头不足；产业层次偏低、结构偏粗、链条偏短，低效益的现象突出，房地产市场运行不稳，优化金融环境还有大量工作要做，经济下行压力依然较大；结构性污染存量大，基础设施欠账多，短期内扭转污染被动局面难度较大；社会事业发展不足，部分群众生活还比较困难，全面建成小康社会的任务十分繁重；政府服务质量、行政效率有待提高，不严不实的问题仍然存在，腐败现象时有发生。对这些问题，我们将采取有力措施，逐步加以解决。

　　二、“十三五”发展总体思路和目标任务

　　党的十八届五中全会勾画了宏伟蓝图，明确了新的发展理念，为我们指明了方向。我国经济发展进入新常态，周期性矛盾和结构性矛盾并存、有效需求和有效供给不足并存、旧动力衰减和新动力成长并存。新乡处在不进则退、转型攻坚的紧要关口，也面临难得的发展机遇。我们要坚定信心，强化担当，努力谱写发展的新篇章。

　　“十三五”工作的指导思想是：高举中国特色社会主义伟大旗帜，全面贯彻党的十八大和十八届三中、四中、五中全会精神，以马克思列宁主义、毛泽东思想、邓小平理论、“三个代表”重要思想、科学发展观为指导，深入贯彻习近平总书记系列重要讲话精神，按照“五位一体”总体布局和“四个全面”战略布局，坚持发展是第一要务，牢固树立和贯彻落实创新、协调、绿色、开放、共享的发展理念，坚持“借势航空港、共建大都市、承担大功能、形成大合力”的工作总思路，发挥区位、科技、山水三大优势，实施产业强市、科技兴市、环境立市三大战略，强化产业集聚区、商务中心区和特色商业区、城乡一体化示范区三大支撑，积极推进“两大一高”战略振兴工程，把提质增效、转变方式、转型升级作为发展导向，着力壮大主导产业，着力培育产业集群，着力发展现代服务业，着力深化改革开放，着力改善生态环境，着力保障和改善民生，建设创新新乡、富裕新乡、文明新乡、绿色新乡，为全省经济社会发展发挥更大作用、作出更大贡献。

　　在确保质量和效益的前提下，我们将发扬滚石上山的精神，自我加压、拉高标杆，力争地区生产总值年均增长8.5%左右，确保主要指标增速在全省位次明显前移、贡献份额明显扩大，确保发展速度快于、发展质量优于、生态环境好于全省平均水平。打造特色鲜明、高端引领的产业创新之城；人才汇聚、活力迸发的创业圆梦之城；绿色发展、水城相依的生态宜居之城；层次更高、融入更广的开放包容之城；普惠均衡、全面小康的富裕和谐之城。实现上述目标要求，要切实增强贯彻五大发展理念的思想和行动自觉，全面提升各项工作水平。

　　——要尊崇创新。创新是新乡过去的主线，也是将来发展的灵魂，决定着我们的兴衰成败。要以创建国家自主创新示范区为载体，更加注重“招才引智”，引导企业成为创新的主体，推动创新资源在新乡集聚。力争到“十三五”末，全社会研发投入占GDP比重达2.5%，引进高层次创新创业团队和人才300个，组建各类创新创业载体30家，扶持9万名大学生就业创业，打造“创客之乡、创业之都、创新之城”。

　　——要注重协调。在加强薄弱领域中增后劲，着力形成平衡发展结构，增强发展的协调性。注重城乡协调发展，按照“一核两带”发展格局，强化“一基本两牵动三保障”，加快农业转移人口市民化，力争到“十三五”末，城镇化率达56%左右。推进三次产业协调发展，到“十三五”末，7大工业产业主营业务收入达5000亿元；服务业增加值达1000亿元以上，占生产总值比重达40%以上；确保粮食总产稳步提升。

　　——要倡导绿色。以刮骨疗毒的态度和决心治理污染，坚持源头控制、末端治理、提高标准和依法严管相结合，努力把环境敏感脆弱期变成生态文明建设的加速期。到“十三五”末，PM2.5浓度下降10%，河流出境断面达标率保持80%以上，森林覆盖率达23%以上。把水系建设作为倒逼产业转型、城市建设和环境保护的重要抓手，用3年时间投入近80亿元，建成“五湖四河二渠”生态水系，新增水面面积1.7万亩。

　　——要厚植开放。大力实施开放带动战略，积极融入“一带一路”。构建立体开放交通体系，推进米字形快速铁路网布局，加快郑新交通一体化，谋划实施环城高速和主城区到各县（市）的快速通道建设，打造中原城市群快速铁路客运北枢纽。积极申建综合保税区，打造举市开放体制。力争“十三五”时期，实际利用内、外资分别达3270亿元和47亿美元。

——要推进共享。坚持民生优先，注重机会公平，保障基本民生，进一步完善就业服务体系、社会保障体系、养老服务体系、住房保障体系和社会事业体系，推进城乡义务教育均衡发展。大力实施精准扶贫、精准脱贫工程，确保全市16.83万贫困人口稳定脱贫。确保城乡居民收入与经济增长同步。让全市人民在共建共享中拥有更多的获得感。

　　各位代表！

　　顾子而不善谋势，得小利而失全盘。在今后发展中，我们将在大势大局中兼顾当前和长远。根据市委的建议，市政府编制了《新乡市国民经济和社会发展第十三个五年规划纲要（草案）》，提请本次大会审议。

　　各位代表！

　　新常态划出新的起跑线，“十三五”的蓝图已经绘就，中央希望中原更加出彩，新乡必须更加有力。我们将脚踏实地、不舍昼夜，始终保持埋头苦干的执著，义无反顾的肝胆，夺取成功的决心，对认准的路子、看准的事情，一张蓝图绘到底、风吹浪打不动摇、不达目的不罢休，努力在新常态的速度换档中实现弯道超越，全力开创新乡各项事业发展的新局面！

　　三、2016年工作安排

　　2016年是“十三五”的开局之年，我们将按照市委总体要求，以开展“招商引智年”“资本运作服务年”“作风建设年”活动为抓手，以提高发展质量和效益为中心，以改革开放和创新驱动为动力，调结构、扩投资、增活力、惠民生、控风险，促进经济平稳健康发展和社会和谐稳定，努力实现“十三五”发展良好开局。

　　今年的主要预期目标是：地区生产总值增长8.5%左右，一般公共预算收入增长7.5%左右，固定资产投资增长15%左右，社会消费品零售总额增长12%左右；城乡居民收入增长与经济增长同步，节能减排完成省定目标。

　　全年着力抓好以下工作：

　　（一）用好支持平台，突出三个转变，强化项目带动。借助国家赋予的各类平台，适应新常态下趋势性变化，提升开放招商、项目建设的层次和水平。

　　借助国家自主创新示范区优势，推动招商引资向招商与引智并举转变。开展“招商引智年”活动，结合国家自主创新示范区建设，以深化院地合作为载体、创新驱动为核心，面向世界和国内500强、行业龙头企业、上市公司、大专院校和科研院所开展招商引智，9月份举办全国知名高校和科研院所走进新乡暨技术对接、经贸洽谈活动，促成一批重大项目和技术落户新乡。争取上级支持，努力将院地合作洽谈会上升到全省乃至全国层面并形成常态，打造我市推进创新、展示形象、招商引智的名片。

　　借助国家产业集聚集群创新发展综合改革试点优势，推动点式招商向链式招商转变。围绕“4325”工程，以产业集聚区为载体，推动产业集群式引进、链接式转移，更加注重通过各类行业协会、商会、中介机构进行招商，大力推进以商招商，每个省级产业集聚区突出发展1个百亿元产业集群，争取开工建设1家投资10亿元以上的龙头企业，实施10个主导产业链项目。打造一批特色鲜明的区中园、园中园，确保原阳家具产业园投产企业达48家以上，平原示范区印刷包装产业园投产企业达10家以上，延津环保产业园入驻企业达26家以上，获嘉装饰涂料产业园入驻企业达16家以上，卫辉食品包装产业园入驻企业达11家以上。推动优质资源向特色园区集聚，力争5年时间形成2家至3家千亿园区。

　　借助国家循环经济示范城市建设地区优势，推动项目建设由数量扩张向质量提升转变。严格环境准入，控制低水平重复建设，在项目规模和质量上实现新提升。尽快启动一批“十三五”重大项目，特别是米字形快速铁路等重大基础设施、赵固低热值煤发电等重大能源设施、生态水系等重大民生工程，促进投资较快增长。坚持重大项目定期观摩等工作机制，确保新乡化纤年产4万吨超柔软氨纶纤维等122个亿元以上项目竣工投产，中原印刷科技产业园二期等242个亿元以上项目开工建设。

　　（二）落实“五大任务”，优化资源配置，强化产业支撑。落实供给侧结构性改革各项要求，优化土地、资本等要素配置，倒逼产业转型升级。

　　加快兼并重组。按照“企业主体、政府推动、市场引导、依法处置”的原则，对有意向进行兼并重组的企业加大引导力度，加快重组进程。一要将兼并重组与培育龙头企业相结合。落实企业兼并重组的各项政策，引导和推进企业兼并重组，促进优势企业做大做强。加大对新航集团、金龙集团、豫飞重工、科隆集团、白鹭化纤、华兰生物等龙头企业的扶持力度，力争经过5年的培育，销售收入超100亿元企业达5家以上。二要将兼并重组与环境保护相结合。对不符合能耗、环保等标准和长期亏损、资不抵债、停产半停产的企业，实行关停并转、兼并重组、破产清算等方式予以出清。整合节能环保、技术改造、循环经济等专项资金，建立以奖代补、奖补后置的机制，出台重点行业提标改造实施方案和“三石”企业重组方案，加强对发电、水泥、化工等涉气、涉水重点排放行业和企业的治理，推动企业技术改造、清洁生产、超低排放，最大限度减少污染。三要将兼并重组与去产能相结合。完善重污染企业退出机制，对没有市场、技术等优势，占用大量土地、污染物排放总量等要素指标的企业，严格按照国家产业政策进行淘汰，推进腾笼换鸟。

　　继续搞好企业服务。坚持领导分包企业等工作机制，做好对涉企收费实行清单管理等工作，抓好财税、科技、金融等涉企政策宣传，确保上级降成本的政策落地见效。定期召开银企对接和人才招聘会，推进企业资本运作和多渠道融资。加强企业培训，打造高素质企业家和管理人才队伍。

　　努力突破新兴产业。以华兰生物、双鹭药业等为龙头，加快中原经济区生物医药产业园等重点项目实施，推动生物与新医药产业整体规模和效益稳步提升。以科隆集团、河南锂动、环宇赛尔、新乡新能等为龙头，整合现有资源，加强与国内外龙头企业对接合作，盘活电池电动车产业资源。以高新区火炬园、红旗区光电信息产业园、平原示范区电子信息产业园等为承接地，加强与北京、上海、深圳等地对接力度，发展电子信息智能终端产业。

　　优化提升支柱产业。以新航集团、豫飞重工等为龙头，推进智能制造，促进高端装备快速发展。以百威英博、娃哈哈、克明面业等为龙头，开发绿色安全的深加工产品，打造国内重要的食品产业基地。以白鹭化纤、香港锦艺等为龙头，加快化纤原料深加工、高档面料、服装产业发展，建设高科技纺织服装产业基地。抓好以新飞电器为龙头的家电产业转型升级和以原阳金祥家具产业园为主导的家具产业达产达效，启动家具物流园和国家级家具检测中心建设，推动现代家居产业加快发展。

　　加快发展现代服务业。强化“两区”生产性服务功能，力争“两区”在建项目达40个以上，完成年度投资40亿元。强力推进新乡国际商务中心建设，支持相对成熟、市场潜力大的专业园区和专业市场纳入“两区”。大力发展大健康、大旅游、大文化产业，推进现代物流、金融等生产性服务业提质发展。加快新乡南太行创建国家5A级景区步伐，打造红色旅游品牌，促进“互联网+旅游”等新模式发展。

　　大力发展现代农业。加强农田水利设施建设，新增高标准粮田40万亩，提升机械化水平，确保粮食产量稳步提升。新增年销售收入10亿元以上农业产业化集群一个。推进国家农业科技园区创建，促进“互联网+”与现代农业深度融合，扩大农民在产业链、价值链中的利益分享比例。

　　（三）融入全省大局，提升比较优势，强化城乡协调。深入贯彻中央城市工作会议精神，提高城市工作的全局性、系统性、持续性、宜居性。

　　提升中心城区辐射带动能力。按照海绵城市标准，提升城市建设品质，中心城区城建重点项目完成年度投资173亿元。一是抓好市政基础设施建设。实施科隆大道东延、牧野大道北延等104个市政基础设施项目，完成年度投资14.8亿元。二是加快生态水系建设。实施中心城区水系连通3年建设计划，完成年度投资29.4亿元。5月底前基本完成赵定河综合治理任务。定国湖要加快推进土方开挖等工作，完成年度投资6亿元。卫源湖要加快局部河段拓宽、截污等工作，完成年度投资5亿元。凤泉湖要完成各项前期工作并开工建设。南湖要力争6月底前竣工。同时加快卫河、人民胜利渠市区段景观改造等项目实施。三是抓好棚户区（城中村）改造。把棚户区（城中村）改造同水系建设和打通断头路相结合，优先实施“沿渠、沿河、沿湖”项目，重点抓好定国村等8个改造项目，新启动755厂区域等21个改造项目，完成年度投资20亿元。同时，抓好万达城市广场、忆通壹世界等重点地产项目建设，完成年度投资110亿元。四是抓好铁西区域开发和大东区建设。坚持统一规划、分步实施，环境优先、基础先行，一期先干、分段推进，加快铁西区域路网、污水管网等基础设施建设。编制大东区空间发展协同总体规划，明确大东区功能定位，加强产业培育，以东带西、形成合力，做大中心城。五是抓好城市管理。落实城市管理“1+4”工作机制，加强监督考核，提高城市精细化、规范化、数字化管理水平。

　　抓好县城发展建设。把县城作为加快城镇化的主阵地、吸纳农业人口转移的主要载体，推动有条件的县（市）打造区域性中心小城市。把产业作为核心支撑，借助“两区”建设，推进旧商业街区和城中村改造，县（市）完成拆迁150万平方米，提升综合承载能力。统筹推进重点镇和新农村建设，改善农村生产生活条件，累计建成300个示范村、500个达标村。

　　促进房地产健康发展。一是以经济手段刺激住房消费。落实税费减免、契税补贴等激励政策，发挥财政资金四两拨千斤的作用，加大金融个贷支持力度。二是用足用好住房公积金。将农民工和个体工商户纳入公积金缴存范围，放宽提取条件、提高贷款额度，发挥公积金对住房贷款支持作用。三是加快棚改安置货币化。制定实施棚户区改造货币化安置实施意见，通过奖补及信贷支持等政策，引导棚改安置由实物安置向货币化安置转变，打通安置保障房和普通商品房通道，消化商品房库存。四是培育新的增长点。落实促进农民进城购房的支持政策，既要通过实施补贴和提供购房服务降低购房负担，又要从创业就业、子女教育、进城落户等方面解决农民工市民化问题。

　　加强交通基础设施建设。把交通项目作为扩大投资的重中之重，实施31个总投资105亿元的交通重点项目，完成年度投资30亿元。推进米字形快速铁路在新乡布局，推进郑州至济南快速铁路和郑济、郑太铁路新乡连接线前期工作。加大新晋高速推进力度，确保鹤辉高速新乡段建成通车，加快推进107国道东移改线、南太行旅游环线前期工作和官渡黄河大桥建设。完成“公交都市”年度创建任务，新增新能源公交车120辆。

　　加快郑新融合。编制郑新融合协同发展总体规划、沿黄经济产业带规划，坚持交通先行、产业链接、服务共享、生态共建，促进与郑州优势互补、良性互动，融入组合型大都市区。支持原阳县、获嘉县等地高水平承接郑州产业转移，把平原示范区作为郑新融合的桥头堡，推进平原示范区电子信息产业园等郑州航空港专属配套园区建设，通过郑新融合，融入“一带一路”发展战略。

　　（四）坚持创新发展，深化各项改革，强化动力转换。加快推进创新创业，抓好重点领域的改革，培育新的经济增长点。

　　以创新增活力。落实科技创新“1+X”工作机制，持续提升创新能力，培育科技协同创新创业中心1家至2家，组建市级以上重点实验室、工程技术研究中心20家，培育国家高新技术企业5家，专利申请量不低于3500件。大力实施质量强市战略，培育10家质量诚信A等工业企业、10个省级以上名牌产品。

　　以创业求活力。再建设一批开放式、低成本、专业化的众创空间，有条件的县（市）、区要规划建设链条式的科技企业孵化体系；加快各类电子商务产业园、科技产业园、大学生创业园建设，促进创新资源向创业者开放共享。鼓励科研院所和企业研发中心在“两区”设立分支机构和成果转化基地。抓好“宽带中国”示范市建设，实施“互联网+制造业”等10个专项行动计划，发展新业态新模式。

　　以改革激活力。落实上级改革任务，抓好简政放权、财税、投融资体制等重点改革事项，同时深挖新乡改革潜力，做好科技协同创新等自选动作，推动改革出成效、出特色、出亮点。以全国农村改革试验区为载体，探索建立城乡一体化体制机制。

　　（五）突出绿色发展，夯实基础能力，强化环境保障。把优化环境作为应对新常态的重要任务来抓。

　　强化生态环境保障。一是大力实施蓝天工程。开展二氧化硫、氮氧化物、扬尘、挥发性有机物等污染物协同控制，对达不到减排目标的企业一律依法停产或限产，确保大气污染指数持续下降，遏制雾霾天气。二是大力实施碧水工程。坚持治水先治污，投资4亿元，完成市区河渠排污口整治、泵站改造建设等43项工程；抓好污水处理设施升级改造，启动东部污水处理厂建设，建成铁西北、大块等污水处理厂并运营，完成年度投资2.8亿元，新增日污水处理能力10万吨。三是大力实施乡村清洁工程。开展“环境示范村、美丽乡村”创建活动。实施土壤保护计划，推进土壤修复。抓好畜禽养殖污染治理，加强农村环境保护。四是加快林业生态建设。启动城市林业生态圈建设工程，打造城市中心区、近郊、远郊配置协调的绿色生态圈，完成林业生态建设19万亩。加快南水北调中线干渠绿化，严格工程标准和质量，3月底前完成全域渠段两侧各100米左右的绿化和5个生态游园建设任务。

　　强化金融环境保障。开展资本运作服务年和金融知识普及年活动，增强社会金融知识，促进金融业发展。加快非法集资案件司法处置进程，加强风险排查，预防新的案件发生，做好稳控工作。完善主办银行制度，推行联合授信管理。严厉打击逃废银行债务等行为，增强银企互信，确保融资主渠道不阻塞。高度重视互联网金融监管，建立既有助于发挥互联网金融优势，又能有效防控风险的监管体系。大力推进新三板挂牌，力争挂牌企业总数突破30家。加大“招行引资”力度，争取更多的股份制银行和地方法人银行在我市设立分支机构。加强与保险机构的合作，引进保险资金。创新金融产品，打造金融超市。做好农信社改制农商行工作，确保完成省政府确定的任务。发挥新型城镇化等基金作用，扩大社会融资，谋划和推进PPP示范项目建设。

　　强化发展环境保障。着力营造良好的干事环境、人才环境、政务环境、法治环境、人文环境，为加快发展提供有力保障。抓好全国文明城市创建、社会信用体系建设等工作，提升全民素质和整体形象。

　　（六）围绕共享发展，弥补工作短板，强化民生改善。坚持共享发展，增强发展的平衡性、协调性、持续性。

　　全面推进脱贫攻坚。大力实施精准扶贫，整合扶贫资源，因人因地施策，提高扶贫实效，再实现3.2万农村贫困人口稳定脱贫。以辉县市、卫辉市、凤泉区为重点，易地扶贫搬迁3680人。抓好黄河滩区居民迁建试点，完成省第一批试点任务，压茬推进省第二批试点，力争再搬迁4299户、15198人。

　　提升教育发展水平。加大教育投入，合理配置教育资源，促进义务教育均衡发展。落实学前教育普及普惠政策，抓好幼师新校区、育才小学东校区、平原幼儿园等重点项目建设；投资1.3亿元，改造农村校舍9.4万平方米；保障进城务工人员随迁子女入学，做好困难学生资助和帮扶工作；推进现代职业教育体系建设，公办高职院校生均财政拨款标准由不低于7000元提高至不低于9000元；支持高校发展，深化校地合作及产学研融合，促进地方经济发展。

　　织密社会保障安全网。推进各类创业孵化园建设，城镇新增就业7万人，农村劳动力转移就业124万人次。扩大社会保险覆盖面，城乡居民社会养老保险参保率保持在99%以上。加大企业职工基本养老保险扩面征缴力度。城镇职工基本医疗保险、城镇居民基本医疗保险政策范围内住院费用支付比例分别提高到80%、76%左右；城镇居民基本医疗保险年人均补助标准再提高60元。提高社会救助保障水平，城乡低保财政补差水平月人均分别提高15元和12元，农村五保对象年集中供养、分散供养标准分别提高200元，达到人均4000元和3000元；发展养老服务业，探索建立医养结合的养老服务新模式。做好中心医院东区医院、市健康管理与康复中心等项目建设，推进结防所、妇幼保健院整体搬迁；抓好县级公立医院改革，促进基本公共卫生服务均等化，控制医疗费不合理增长，建立和谐医患关系；新农合财政补助标准再提高30元。做好公租房与廉租房并轨运行后廉租房的转换工作，完成省政府下达的保障性住房各项任务。

　　统筹各项事业发展。抓好平原体育综合训练中心建设，加快向全民免费开放公共文化体育设施进程。落实全面两孩儿政策。支持群团组织更好开展工作。认真落实党的民族、宗教和侨务政策。深入开展双拥共建，支持国防和军队建设，推进军民融合深度发展。统筹抓好防震减灾、统计、史志、外事、侨务、档案、测绘、人防等工作，促进各项事业全面进步。

　　维护社会和谐稳定。加快安全生产隐患排查治理长效机制建设，加强食品药品监督管理，强化源头治理和专项整治，防止重大事故发生。加强和创新社会治理，做好信访、仲裁、人民调解、行政复议和法律援助等工作。完善立体化社会治安防控体系，防范和化解各类不稳定因素。提高应对群体性事件、突发事件的能力水平，维护社会稳定。

　　（七）抓好自身建设，推进作风转变，加强反腐倡廉。开展作风建设年活动，加强政府自身建设，提升履职能力。

　　全面依法行政。健全科学民主决策机制，自觉接受市人大法律和工作监督、政协民主监督、新闻舆论监督和社会公众监督。做好地方立法工作，完善政府部门权力清单运行与监督管理机制，确保群众的知情权、参与权、表达权和监督权。打造职能科学、权责法定、执法严明、公开公正、廉洁高效、守法诚信的法治政府。

　　狠抓工作落实。践行“三严三实”，把各项部署变成具体项目、具体措施，脚踏实地，凝神聚力，一件事一件事抓落实，积小胜为大胜。各级各部门要站位全局，不上交矛盾、不推诿扯皮，坚决反对遇事讲条件、讲困难、讲客观。聚焦监督执纪问责，开展懒政怠政为官不为问责年活动，建立完善奖罚分明的结果运用机制，营造干事创业的浓厚氛围。

　　加强反腐倡廉。坚持纪在法前、纪严于法，尊崇党章，严格执行准则和条例，看住重要节点，紧盯“四风”新病源，用铁的纪律整治各种顶风违纪行为，加大通报曝光和问责追责力度。坚持无禁区、全覆盖、零容忍，坚决惩治贪污腐败分子。畅通信访举报渠道，坚决查纠基层贪腐和执法不公等问题。推动落实“两个责任”，完善反腐倡廉制度，把权力关进制度的笼子。

　　各位代表！

　　蓝图已经绘就，实干铸就辉煌。让我们紧密团结在以习近平同志为总书记的党中央周围，在省委、省政府和市委的坚强领导下，发扬“说了算、定了干、再大困难也不变”的精神，团结一心、埋头苦干，持续而为、久久为功，再创新乡的新辉煌，努力在全省发挥更大作用、作出更大贡献。
